# PDLIM2 is highly expressed in Breast Cancer tumour-associated macrophages and is required for M2 macrophage polarization

**DOI:** 10.3389/fonc.2022.1028959

**Published:** 2022-11-30

**Authors:** Orla T. Cox, Neil O’Sullivan, Emilie Tresse, Stephanie Ward, Niamh Buckley, Rosemary O’Connor

**Affiliations:** ^1^ Cell Biology Laboratory, School of Biochemistry and Cell Biology, BioSciences Institute, University College Cork, Cork, Ireland; ^2^ School of Pharmacy and Patrick G. Johnson Centre for Cancer Research, Queens University, Belfast, United Kingdom

**Keywords:** Triple Negative Breast Cancer (TNBC), PDLIM2, M2 macrophage, cell differentiation, immune infiltration

## Abstract

The PDZ-LIM domain-containing protein 2 (PDLIM2) regulates cell polarity and the protein stability of key transcription factors in epithelial and hemopoietic cells. We previously reported that PDLIM2 is more highly expressed in Triple Negative Breast Cancer (TNBC) than in other breast cancer types or normal breast tissue. In the course of the TNBC study, it was noted that PDLIM2 was highly expressed in the stroma of PDLIM2-expressing tumours. Here, we investigated the phenotype of these stromal cells and whether any infiltrating immune population was linked to PDLIM2 expression. We found that high PDLIM2 expression in breast tumours was associated with higher levels of infiltrating M2 macrophages, but was not associated with infiltrating T cell sub-populations. We then tested whether PDLIM2 contributes to macrophage differentiation or function by using cultures of bone marrow-derived macrophages from wildtype and *Pdlim2* knockout mice. This demonstrated that PDLIM2 is required for naïve macrophage migration and for the full adoption of IL-4-induced M2 polarization, including expression of M2 phenotypic markers, cell adhesion and cell migration. TLR4-, TLR3- or IFNγ-induced M1 macrophage activity was less dependent on PDLIM2. Finally, analysis of publicly available breast cancer datasets showed that high PDLIM2 expression is associated with increased M2 macrophage infiltration. We conclude that PDLIM2 expression influences the tumour associated stroma and, in particular, M2 macrophage infiltration that may contribute to the progression of TNBC or other subsets of breast cancer.

## Introduction

Better defining the signalling interactions between epithelial cells and immune cells in the cancer tumour microenvironment is essential for understanding cancer progression and for selecting optimal strategies for therapy, including the deployment of immunotherapies that target the immune regulatory pathways (1–3). In breast cancer this is particularly important for the TNBC subset, which is heterogenous, aggressive and is also increasingly associated with the infiltration of immune cells (4, 5).

PDLIM2 is a PDZ and LIM domain containing protein expressed in both epithelial and hemopoietic cells that has a well-defined role in regulating the activity of STATs, NFkB and beta catenin (6–11). Repression of PDLIM2, typically through epigenetic mechanisms, is associated with tumourigenesis in several tissues including lung, breast and colon [reviewed in (10, 12)]. On the other hand, elevated expression of PDLIM2 expression is observed in aggressive and metastatic cancers, including prostate cancer and TNBC (11–13). In this scenario, suppression of PDLIM2 in cell and murine models has been shown to impair polarized cell migration and metastatic potential of tumour cells *in vitro* and *in vivo* (14).

While it is still not well understood how PDLIM2 levels or activity are regulated in different tissues or during cancer development or progression, its regulation of key oncogenic signalling pathways suggests strong potential to modulate the tumour microenvironment, at both the level of the tumour cells and associated infiltrating immune cells. Knockout mouse studies have shown that PDLIM2 is required for normal T lymphocyte differentiation, and the absence of PDLIM2 skews differentiation towards Th1 and Th17 subsets (15). In differentiated macrophages, PDLIM2 accumulates in the cytoplasm, where it is required for their adhesion and for NFκB regulation (16). Moreover, PDLIM2 suppression by reactive oxygen species (ROS) in alveolar macrophages has been associated with the polarization/activation of pro-tumourigenic macrophages in lung cancer (17).

We previously reported that PDLIM2 expression was elevated in TNBC compared with other breast cancers or normal breast tissue, and that this was associated with amplified cell adhesion and beta catenin signalling (11). However, it has not been established whether there is a clinical consequence of high PDLIM2 expression in TNBC or other breast cancers. To address this further, we interrogated the high expression of PDLIM2 that was observed in the stroma of this breast cancer cohort.

Our findings demonstrate that high PDLIM2 expression in breast cancer is predominantly associated with higher levels of CD163- and CD206-positive macrophages that likely represent the M2 subset, whereas the expression of T lymphocytic markers is not uniquely associated with PDLIM2 expression. Using bone marrow derived macrophages from wildtype and *Pdlim2* knockout mice, we determined that PDLIM2 is required for macrophage migration and the full adoption of the M2 macrophage phenotype. Analysis of METABRIC and TCGA breast cancer datasets *via* immune deconvolution methods further demonstrated an association of PDLIM2 mRNA and protein expression with M2 macrophage infiltration. We conclude that PDLIM2 influences the activity of this population of macrophages in breast cancer.

## Materials and methods

### Chemicals and reagents

IFNy (#315-05) and IL-4 (#214-14) and MCP-1 (#250-10) were purchased from Peprotech, (London, UK); Alexa 488- and Cy3-conjugated secondary antibodies (#711-545-152 and 715-165-150) IRDye® 680 (#926-68070 and #925-32211) were purchased from Jackson ImmunoResearch Laboratories (Cambridgeshire, UK). All other reagents were from Merck (Dublin, Ireland), unless otherwise noted.

### Breast cancer tissue samples

The Breast Cancer Tissue Microarrays (TMAs) were constructed at the *Northern Ireland Biobank (NIB)* from Formalin-fixed paraffin-embedded primary tumor blocks as previously described (11, 18). Each tumor sample was represented by three independent cores. Breast Cancer subtypes were determined from biomarker expression, and classified according to St Gallen International Expert Consensus (19) as: Luminal A (ER and/or PR positive); Luminal B (ER and/or PR positive and HER2 negative (HER2-) or HER2 overexpressed/amplified (HER2+); HER2 enriched (non-luminal, ER and PR negative and HER2 overexpressed/amplified); Basal-like/Triple negative (ER, PR and HER2 negative). Cases were diagnosed in Northern Ireland from 1997 to 2009 with ethical approval granted by the Northern Ireland Biobank (20) and faculty research ethics committee (NIB15-0156, NIB12-0043, NIB12-0017 and MHLS 20_43).

### Antibodies

Antibodies used in this study that were purchased from Cell Signalling Technologies (Danvers, MA, USA) include those that detect: Phospho-Akt/non-phospho Akt (#4060/#2920), phospho-p38/non-phospho p38 (#9211/#9212), phospho-Erk/non-phospho Erk (#4377/#4696), phospho-JNK/JNK (#9251/#9252), phospho-JAK1 (#3331), JAK2 (#3230), phospho-STAT1 (#7649), phospho-STAT3 Y705 (#9145), phospho-STAT6 (#9361), phospho-IκBα/non-phospho-IκBα (#9241/#9242), phospho-p65/non-phospho p65 (#3033/#8242), iNOS (#13120) and IL-1b (#12242). Antibodies from Santa-Cruz Biotechnology (Santa Cruz, CA, USA) include those targeting HO-1 (sc-136960), IRF4 (sc-28696), α-tubulin (sc-5286), IRF3 (sc-33641). Anti-HIF-1α antibody (#A300-286A) was from Bethyl laboratories, (Montgomery, TX, USA), E-Cadherin (#610181) was purchased from BD Biosciences, (Wokingham, England, UK). Mouse and rabbit anti-PDLIM2 antibodies have been described previously (14).

### Immunohistochemistry staining of TMAs and scoring of biomarkers

All immunohistochemistry (IHC) was performed in a hybrid laboratory (Precision Medicine Centre of Excellence), awarded UK Clinical Pathology Accreditation. Sections were cut from the TMA blocks for haematoxylin and eosin staining and IHC for the indicated biomarkers as previously described (11, 18, 22). Briefly, 4 μm sections for IHC were cut on a rotary microtome and dried at 37 °C overnight. IHC was performed on automated immunostainers (Leica Bond-Max, Milton Keynes, UK or Ventana BenchMark, Tucson, AZ). Each biomarker was initially validated on carefully selected control tissues. Antigen-binding sites were detected with a polymer-based detection system (Leica Biosystems UK, Cat. No. DS9800 or Ventana USA Cat. No. 760–700 and Cat. No. 760-500). Sections were incubated with diaminobenzidine (DAB) for detection, counterstained with haematoxylin, and tape mounted using a Sakura Autostainer (Sakura Finetek Europe, Rijn, Netherlands). All slides were scanned on an Aperio AT2 digital scanner (Leica Biosystems, Milton Keynes, UK) at ×40 magnification. Quality control checks ensured images were captured without digital scanning artefacts that might interfere with downstream analysis. Staining was scored based on negative (0), low (1), moderate (2) or high (3) expression scoring system (11), blinded to patient clinicopathological and outcome data, and consensus scores from tissue sample triplicate cores were determined.

### *Pdlim2^–/–^* mice and preparation of BMDM

*Pdlim2* ^–/–^ C57Bl/6J mice were generated from the previously described Balb-c *pdlim2-/-* mice (8) by backcrossing at least eight times with with C57Bl/6J mice. All animals were housed and cared for in compliance with protocols and procedures approved by the Animal Experimentation Ethics Committee of University College Cork and the Health Products Regulatory Authority (Project Authorisation Number: AE19130/P077). Bone marrow cells were isolated from the femurs of *Pdlim2* Wild Type (+/+) or knock-out (-/-) mice at 8-12 weeks, and differentiated into Bone Marrow-Derived Macrophages (BMDM) as described (23), and cultured in DMEM supplemented with 10% Foetal Bovine Serum and 15% L929 Cell Conditioned medium (LCCM). Non-adherent cells were removed 7 days post-isolation. The remaining adherent macrophages were detached using 0.5 mM EDTA and re-seeded onto tissue culture plates. All experiments were performed within 11 days of isolation to avoid spontaneous macrophage differentiation.

### M1 and M2 macrophage polarization

Polarized BMDM were designated according to the stimulants used for polarization, based on nomenclature adapted from (24). Conditions for Lipopolysaccharide (LPS) stimulation were first optimized using (10 or 100ng/ml) over time (0.5-72hr) in freshly isolated PDLIM2+/+ BMDM to generate MΦ_LPS_. LPS was subsequently used at 100ng/ml and cells for 6hr stimulation (short term response) or 24hr (long term response). Polyinosinic: Polycytidylic acid (PI:C) was used at 50µg/ml, IFNy at 10ng/ml and IL-4 at 10ng/ml for 6 and 24hr stimulation to generate MΦ_PIC,_ MΦ_IFN,_ MΦ_IL4_, respectively. Methods were based on those previously described (25–29). Briefly, BMDM were seeded in complete growth medium at 1.5-2x10^6^ per 10cm plate, for preparing protein samples, at 5-6x10^5^ per 6cm plate for RNA extraction, or at 3x10^4^/well of 8-well slide (lab-tek, (Thermo Scientific Nunc, Cork, Ireland) for immunofluorescence. Following stimulation, cells were harvested using 0.5mM EDTA or directly in lysis buffer using a cell scraper.

### RNA extraction and quantitative PCR

RNA was obtained from BMDM using Genejet RNA extraction kit (Fisher Scientific, Ireland) and cDNA was synthesised using the Quantitect Reverse Transcription kit (Qiagen, Crawley, UK). Quantitative PCR was carried out using the LightCycler instrument (Roche Molecular Biochemicals, East Sussex, UK) using FastStart Essential DNA Green Master kit (Roche Diagnostics, West Sussex, UK, Cat#6402712001). The 2^-ΔΔ CT^ method was used to analyze data and determine relative mRNA expression levels normalized to the housekeeping gene 18S as previously described (14). The data are presented as relative mRNA levels to those of the genotype naïve BMDM controls. Primers were synthesized by Integrated DNA Technologies (IDT Europe, Leuven, Belgium). 5’-3’ Primer sequences are listed below for Forward (For) and Reverse (Rev) primers for each gene.

**Table:** 

Il-12b	*For : AGAAAGGTGCGTTCCTCGTAG, Rev : AGCCAACCAAGCAGAAGACAG*
Il-1β	*For: CAACAAACAAGTGATATTCTCCATG, Rev : GATCCACACTCTCCAGCTGCA*
CD206	*For: TCTTTTACGAGAAGTTGGGGTCAG, Rev: ATCATTCCGTTCACCAGAGGG*
CD38	*For: TGAAAACTGTCCCAACAACC Rev: AACTAAAGACTTCCACACTTCC*
CXCL10	*For : GACGGTCCGCTGCAACTG, Rev: CTTCCCTATGGCCCTCATTCT*
CCL5	*For: CTGCTGCTTTGCCTACCTCT, Rev: TCTTCTCTGGGTTGGCACAC*
iNOS	*For: CCCTCCTGATCTTGTGTTGG, Rev: GGCAGTGCATACCACTTCAA*
IRF5	*For: AATACCCCACCACCTTTTGA, Rev: TTGAGATCCGGGGTTTGAGAT*
SOCS3	*For: CTTTTCTTTGCCACCCACGG, Rev: CTTTTCTTTGCCACCCACGG*
YM1	*For: TCAACGGTTTTTCCACAGTGC, Rev: TCCCAGCTGGTACAGCAGAC*
TNFα	*For: TGTCTACTCCTCAGAGCCCC, Rev: TGAGTCCTTGATGGTGGTGC*
IL-6	*For: GAGGATACCACTCCCAACAGACC, Rev : AAGTGCATCATCGTTGTTCATACA*
FIZZ1	*For: CTGCTACTGGGTGTGCTTGT, Rev: GCAGTGGTCCAGTCAACGAG*
IFNγ	*For: TTCTTCAGCAACAGCAAGGCGAA Rev : TGAATGCTTGGCGCTGGACCTG*
18S	*For : AACCCGTTGAACCCCATT; Rev: CCATCCAATCGGTAGTAGCG*

### Cell lysis and western-blotting

Total cellular protein extracts were prepared as described previously (7, 14, 16). Briefly, whole protein extracts were prepared using RIPA lysis buffer with phosphatase and protease inhibitors and lysates were resolved by SDS-PAGE. Samples were probed for protein expression by western blotting using the Odyssey Image scanner system (LI-COR Biosciences, Cambridge, UK), as previously described (11, 14, 30). The approximate protein molecular weights in kilodaltons are indicated on the left of each western blot panel.

### ELISA and nitric oxide assays

Supernatants were collected from BMDM following activation, and subsequently subjected to analysis using the Griess Assay system, according to manufacturer’s instructions (Promega, Southampton, UK, #G2930). This assay measures NO2^-^, which is a breakdown product of NO production and indicative of NO production by the macrophages.

ELISAs were performed using the Mouse IL-1β platinum ELISA and Mouse TNF-α Instant ELISA kits (#BMS6002, #BMS607/2INST, Thermofisher Scientific, Biosciences, Dublin, Ireland), following the manufacturer’s instructions. Signals were measured using a SpectroMax plate reader spectrophotometer coupled to SoftMaxPro Software (Molecular Devices, Wokingham, UK).

### Cell adhesion and migration assays

BMDM migration was analyzed by Transwell migration and agarose spot chemotaxis assays. For each assay, BMDM were first cultured on 10cm plates and stimulated with LPS or IL-4 for 24h. Cells were washed with PBS and harvested using 4mg/ml lidocaine (Sigma, Dublin, Ireland). Transwells with 0.8 µm filters were used (Corning Life Sciences, UK). Cells were seeded at 5x10^5^ in triplicate onto the upper well of transwell chambers. Lower chambers contained medium supplemented with Monocyte Chemoattractant Protein-1 (MCP-1, 20ng/mL; Peprotech) as chemo-attractant. After 24h, cells were fixed, stained with 0.1% crystal violet, scanned and quantified using the Odyssey system (LI-COR Biosciences) as previously described (14, 30). Data are presented as relative migration using arbitrary units, with migration of PDLIM2+/+ minus chemoattractant set as 1 under all conditions.

Chemotaxis agarose spot assays were performed as described in (31) using 20ng/ml MCP-1 as the chemoattractant inside the spot. Cells were seeded at 3x10^5^ per well of a 48-well plate for agarose spot assays and after 24hr cells were photographed using a Nikon Eclipse TE300 inverted Microscope with a Hamamatsu C11440 ORCA-flash 4.0LT Digital Camera and Nikon NIS-Elements imaging software AR version 4.50 (Aquilant Scientific, Dublin, Ireland).

For cell adhesion assays, cells were seeded at 1.5x10^4^ per well of a 96 well plate and allowed to adhere for 30min to measure initial adhesion or for 6h to measure sustained adhesion. Cells were then washed and stained with crystal violet, which was quantified with an Odyssey Image scanner system (LI-COR Biosciences) as previously described (14, 32).

### Immunofluorescence

Immunofluorescence was performed as described previously (11, 30). Briefly, BMDM were cultured on glass 8-well chambers, washed, and then fixed with 4% paraformaldehyde. Cells were incubated with primary antibodies overnight followed by Alexa 488- or Cy3-conjugated secondary antibodies for one hour, and/or TRITC-phalloidin and nuclei visualized with Hoechst dye. Cells were photographed at 40X magnification using a Nikon Eclipse E600 microscope, equipped with a SPOT digital camera and SPOT software version 4.6 (Diagnostic Instruments Inc, Sterling Heights, MI, USA).

### *In silico* profiling of tumour infiltrating immune cells

The Molecular Taxonomy of Breast Cancer International Consortium (METABRIC) (33) dataset was downloaded from cBioPortal (34) on March 3^rd^, 2022. The Cancer Genome Atlas (TCGA) PanCancer Atlas breast cancer (BRCA) dataset was downloaded from cBioportal on April 6^th^, 2022. Analysis of immune cell infiltration of the METABRIC microarray and TCGA-BRCA RNA-seq datasets was conducted using CIBERSORT, a computational algorithm which estimates cellular composition of complex tissues from bulk gene expression data (35). CIBERSORT estimation scores for the METABRIC dataset were determined using CIBERSORTX (36). Gene expression values for the METABRIC cases (n= 1904) were converted to non-log space, and missing values were imputed *via* K nearest neighbours (kNN) imputation (K = 10), to allow for the CIBERSORT requirement of inputs being in non-log space and no missing entries. The following settings were used for CIBERSORTX: LM22 signature matrix, Batch correction B-mode, Quantile normalization disabled, Absolute mode, 100 permutations. Of the obtained deconvolution results, successfully deconvoluted samples were identified by selection of samples with a p-value of ≤ 0.05.

For the TCGA-BRCA cohort, CIBERSORT estimation scores were obtained from TIMER2.0, which provides collated immune cell estimation values for all TCGA samples across various deconvolution algorithms (37).

All breast cancer cases with immune estimation data within the METABRIC (n = 1894) and TCGA-BRCA (n = 1079) cohorts were stratified into tertiles, rounded to the nearest whole number. These tertiles represent PDLIM2_low_, PDLIM2_Mod_, and PDLIM2_High_ groups as follows: METABRIC total breast cancer (low: n=631, mod n=632, high: n=631); TCGA total breast cancer (low: n=360, mod: n=359, high: n=360). TNBC cases were identified from the criteria described by Lehmann et al. (38), which uses multi-omic evaluation of ER, PR, and HER2 status, in combination with clinical IHC and FISH assessments. Of the cases with deconvolution data, the cases were grouped as follows: METABRIC TNBC (low: n=107, mod n=106, high: n=107); TCGA-BRCA TNBC (PDLIM2 low: n=63, mod: n=64, high: n=63).

CIBERSORT Immune infiltration scores for total CD4+ T cells were determined by the aggregation of scores for CD4+ naïve, CD4+ memory resting, CD4+ memory activated, follicular helper, and regulatory T cells, as previously described (39).

### Statistical analysis

Data from breast tissue immunohistochemistry were analyzed for statistical significance using Fisher’s exact test. In experiments with cultured BMDM data were analyzed for statistical significance using Student’s *t* test to compare samples. Data are presented as mean with the Standard Deviation (SD) or Standard Error of the Mean (SEM) and p values are included where there was a statistically significant difference; *p* < 0.05 was deemed significant, and graded *p* values are represented as follows: *p < 0.05, **p < 0.005, and ***p < 0.0005. Statistical analysis of immune deconvolution data was performed using two-tailed Mann-Whitney test, to compare the mean ranks of the stratified groups. Scatter plots of deconvolution results are presented with median values.

## Results

### PDLIM2 is expressed in the stroma of breast cancer tumours

**Figure 1:**
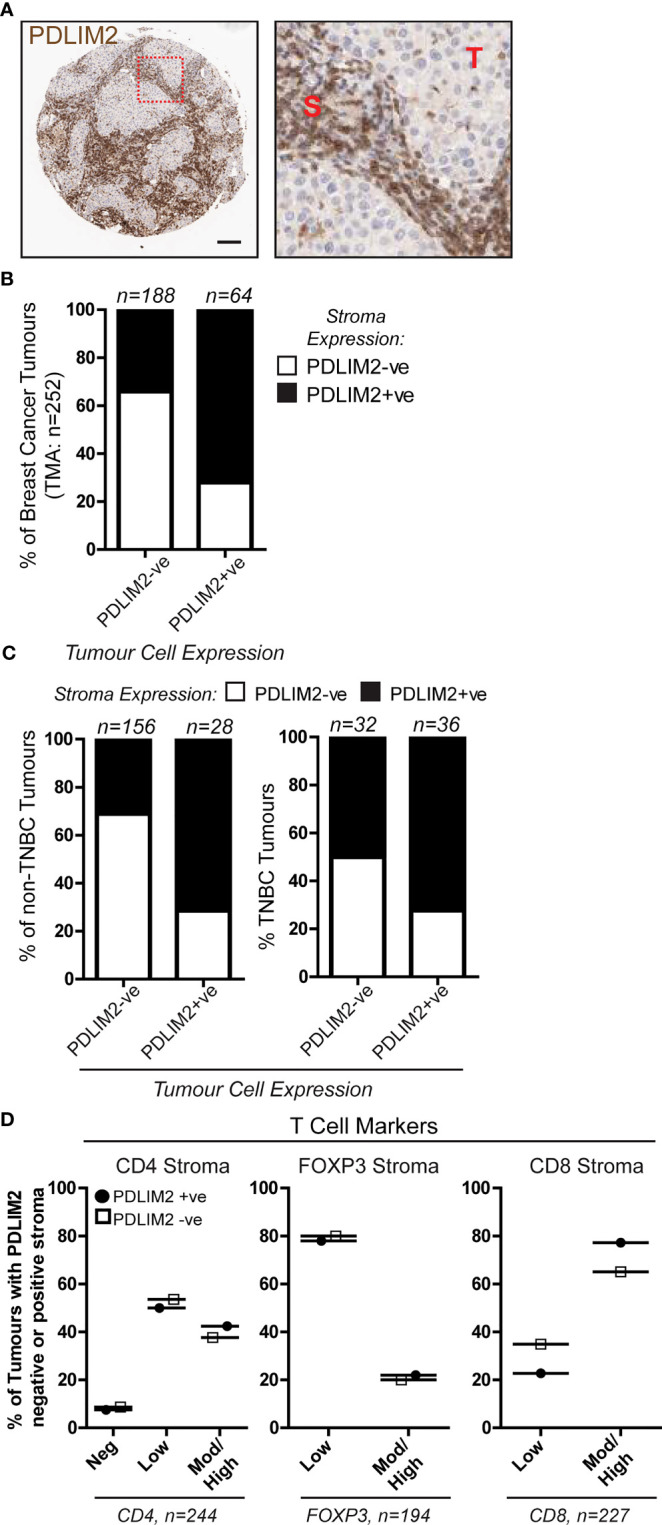
PDLIM2 expression in PDLIM2-negative and -positive breast cancer tumour-infiltrating stroma is not associated with any particular T cell population. A Breast Cancer TMA from a cohort of approximately 250 samples from the Northern Ireland Biobank (NIB) was stained for PDLIM2 protein expression by immunohistochemistry (11). **(A)**: Representative micrographs of PDLIM2 IHC staining in breast tumour (Her2-enriched). Right panel shows zoomed area to illustrate PDLIM2 staining in tumor cells (T) and stromal cells (S). Scalebar represents 100µm. **(B, C)**: Quantification of PDLIM2 negative (-ve) or positive (+ve) expression in stroma of all breast cancer cores **(B)**, or data shown separately for non-TNBC (left panels) and TNBC tumours (right panels) across the cohort **(C, D)**: Expression of T cell markers scored as negative, low, moderate (mod) or high in PDLIM2-ve or +ve stroma. Data are presented as percentage of each marker expression score within PDLIM2 -ve or +ve groups.

We previously observed in a breast cancer TMA cohort that PDLIM2 is expressed in 40-60% of the infiltrating stroma of tumours (**Figure 1A, B**, 11). In this TMA (approximately 250 cores), more than 70% of PDLIM2-positive tumours exhibited PDLIM2 expression in the stroma compared to PDLIM2-negative breast tumours where PDLIM2 was present in only 30% of stromal cells (**Figure 1B**). When data for TNBC tumours was separated from other breast cancer subtypes it was observed that PDLIM2 expression was overall higher in the stroma of TNBC (62% compared with 37% stromal expression in non-TNBC tumours (**Figure 1C**), including higher expression in PDLIM2-negative TNBC tumours (circa 50%), than in the other breast cancer subtypes (circa 30%), (**Figure 1C**) and (11).

To determine whether PDLIM2 is associated with a particular immune cell subset, we analyzed the phenotype of the tumour-infiltrating cells, first focusing on T cell populations. The level of staining for each T cell marker (CD4, CD8 and Foxp3 for T reg) was scored as negative, low, moderate/high, and this was correlated with the score for PDLIM2 staining in the tissues. This demonstrated that in the stroma of PDLIM2-positive and PDLIM2-negative tumours the percentages of CD4+, CD8+ or Foxp3+ T cells present in all tissues and stroma were similar, regardless of PDLIM2 expression (**Figure 1D**, **Supplementary Figure 1A**). When TNBC cores were analyzed separately, similar levels of staining for each T cell subset were observed in PDLIM2-positive and -negative tumours and stroma (**Supplementary Figures 1B, C**).

Taken together, these data demonstrate that in all breast tumours that exhibit expression, PDLIM2 is also highly expressed in the stroma, while TNBC displays high PDLIM2 in the stroma of all tumours. However, high PDLIM2 expression was not significantly associated with either of the CD4+, CD8+ or Fox3p+ T cell sub-populations that were detected in the stroma of these tumours.

### M2 Macrophages are predominant in a PDLIM2-positive tumour microenvironment

**Figure 2:**
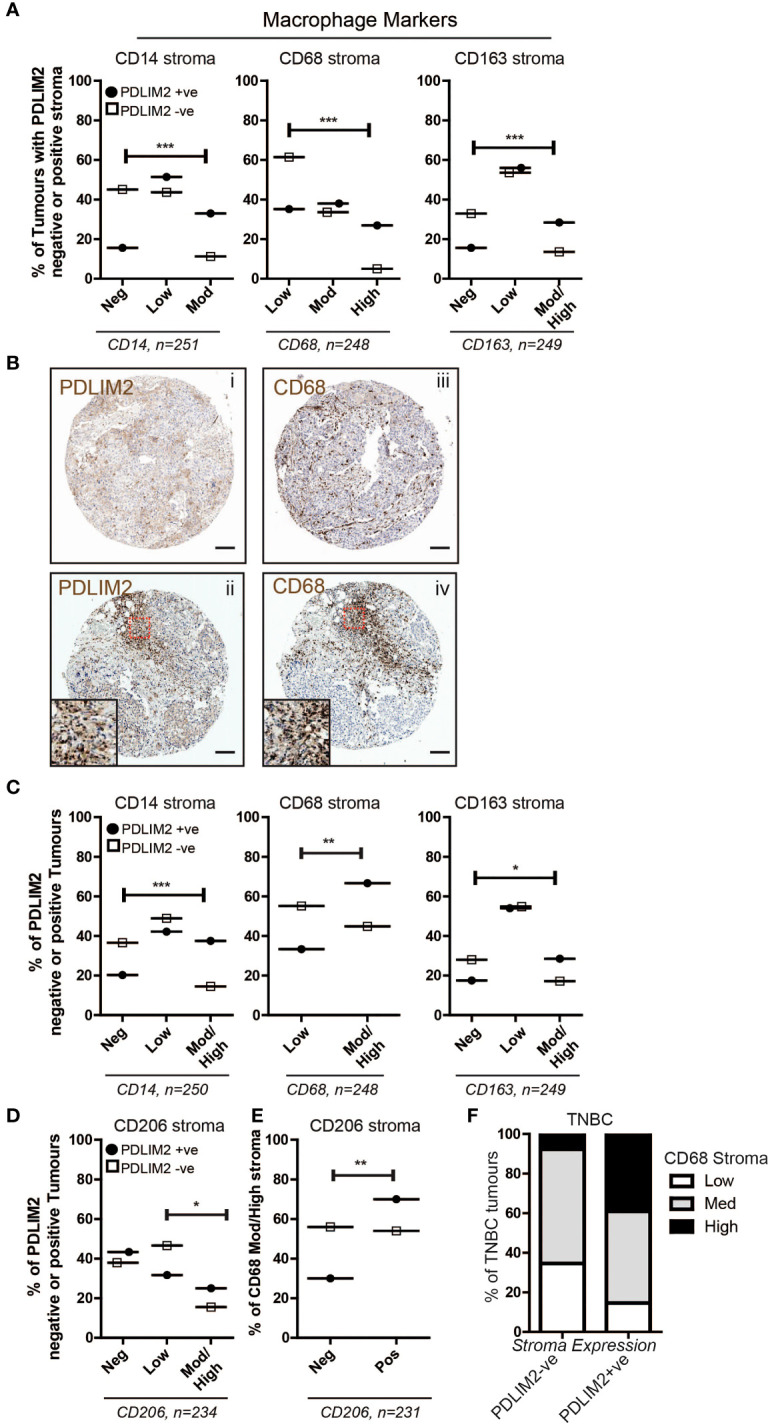
PDLIM2 expression is associated with the M2 macrophage population in the breast tumour microenvironment. **(A)**: Expression of Macrophage markers CD14, CD68 and CD163 in the breast cancer TMA scored as negative, low, moderate or high in PDLIM2-ve or +ve stroma. Data are presented as a percentage of each marker expression score within PDLIM2-ve or +ve groups (from 3 cores). Fisher’s exact test: *p<0.05, **P< 0.005, p<0.0001. **(B)**: Representative micrographs of IHC staining for PDLIM2 (i and ii) and CD68 (iii and iv) in serial sections of TNBC tumours. Insets in panels ii and iv illustrate significant overlap of high infiltration by CD68-positive macrophages in PDLIM2+ve stroma. Scalebars represent 100µm. **(C)**: Expression of Macrophage markers in the stroma of breast TMA tumours with PDLIM2-ve or +ve tumour cells, as described for stroma analyses in **(A, D)**: Quantification of expression of the M2 marker CD206, in PDLIM2-ve or +ve tumours. **(E)**: CD206 expression in CD68-rich PDLIM2-ve or +ve stroma. **(F)**: Expression of macrophage marker CD68 in TNBC tumours with PDLIM2+ve or PDLIM2-ve stroma. Representative IHC images capturing negative and positive staining patterns for macrophage markers are shown in **Supplemental Figures 1G–I**.

Our next objective was to investigate macrophage populations in the breast TMA by first analyzing the expression of CD14 (monocytes), CD68 (all macrophages), and CD163 (M2 macrophages), and further stratifying the expression of these markers within PDLIM2-positive and -negative tumours. The results demonstrated significantly higher numbers of CD14+ and CD68+ cells in the tumour infiltrate of PDLIM2-positive tumours and in PDLIM2-positive stroma than in either PDLIM2-negative tumours or stroma (**Figures 2A–C**). Indeed, within serial sections of PDLIM2-positive tumours, there was considerable overlap in the profile of CD68 and PDLIM2 stromal staining (**Figure 2B**), while the expression of CD68 within the tumour was similar in all cores (**Supplementary Figure 1D**).

To further establish the phenotype of the tumour-associated macrophages (TAMs) that express PDLIM2, we analyzed the percentage of PDLIM2-positive or -negative tumours that expressed low or medium/high levels of CD163. It has previously been observed that M2-like Macrophages including TAMs, may express higher levels of CD163 than M1-macrophages [reviewed in (40)]. We also assessed a key M2 marker, CD206 (24). Higher scores for moderate/high expression of CD163 were observed in PDLIM2-positive stroma and intra-tumour infiltrates than in PDLIM2-negative tumours, while the scores for low CD163 expression were similar (**Figures 2A, C**, **Supplementary Figure 1D**). CD206 expression was overall similar in PDLIM2-positive and PDLIM2-negative stroma (**Supplementary Figure 1E**). However, CD206 was expressed at significantly higher levels in the stroma of breast cancers where the tumour cells also expressed PDLIM2 (**Figure 2D**). Further analyses demonstrated that of the tumours with high macrophage infiltration (CD68 moderate/high scoring stroma), 70% of these were CD68+/CD206+, compared with PDLIM2- negative stroma where 54% of macrophages expressed CD68 and CD206 (**Figure 2E**). These results indicate that M2 macrophages are more abundant in PDLIM2-positive tumours and stroma.

Since PDLIM2 is significantly more highly expressed in TNBC than in other breast cancer types (11), we analyzed the phenotype of macrophages present in TNBC cores. This demonstrated higher CD68 in the stroma of TNBC PDLIM2-positive tumours than in PDLIM2-negative tumours (**Figure 2F**). Importantly, out of all PDLIM2-positive tumours, 40% expressed high levels of CD68 compared to less than 10% of the PDLIM2-negative tumours (**Figure 2F**). More than 85% of PDLIM2-positive TNBC tumours expressed moderate or high CD68 expression compared to 65% of the PDLIM2 negative tumours (**Figure 2F**). CD206 was significantly higher in TNBC tumours with PDLIM2-positive stroma than in those with PDLIM2-negative stroma, while CD163 was higher, but not quite reaching significance (**Supplementary Figure 1F**, left panels). Expression levels of CD206 and CD163 were higher in the stroma of PDLIM2-positive than in PDLIM2-negative TNBC tumours, but these differences were not significant (**Supplementary Figure 1F**, right panels).

Overall, we conclude that M2 macrophage infiltration is enhanced in PDLIM2- positive tumour stroma, with CD163+ and CD206+ macrophages more strongly represented in these tumours than in PDLIM2-negative stroma. Moreover, high PDLIM2 expression with M2 macrophage enrichment is more evident in the stroma of TNBC than in other breast cancer subtypes.

### PDLIM2 is required for naïve macrophage migration and TLR4-induced IL-1β secretion and NO production, but not for TLR3 or IFNyR responses

Our analysis of PDLIM2 expression in breast tumour tissue suggests that its presence may favour the expansion of the M2 macrophage population. PDLIM2 has a known function in immune cell differentiation and macrophage function (15, 16). Therefore, we next investigated whether PDLIM2 is required for the differentiation of macrophages to the M1 (pro-inflammatory) or M2 (wound-healing) subtypes using primary bone marrow-derived macrophages (BMDM) from *Pdlim2* Wild Type (+/+) or knock-out (-/-) mice.

**Figure 3:**
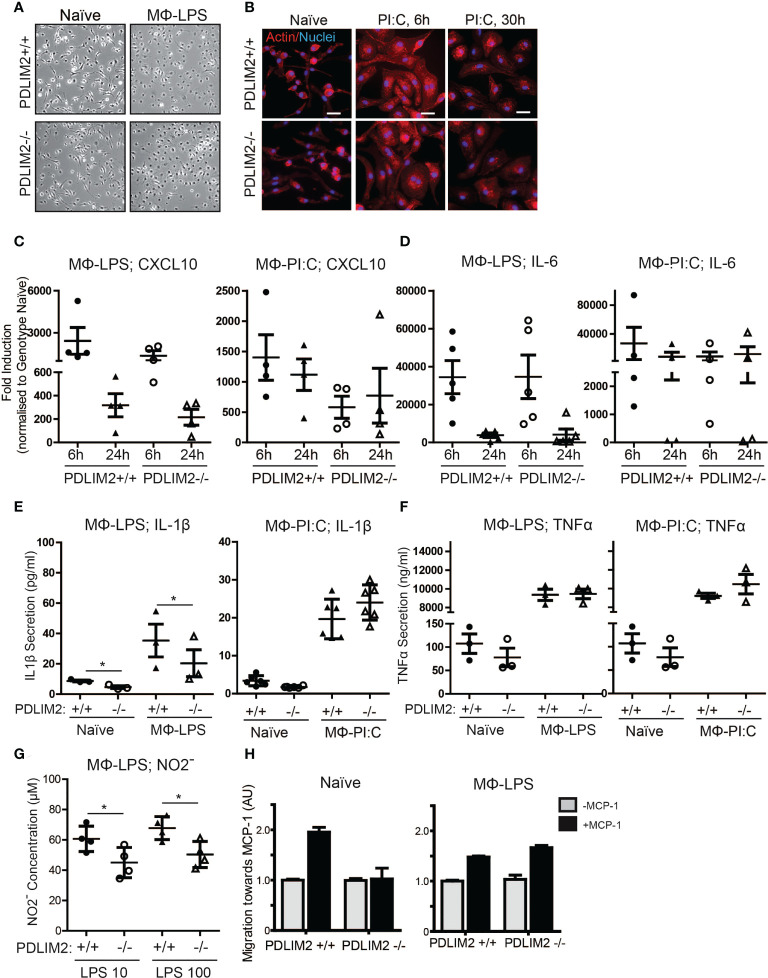
PDLIM2 is required for key TLR4-mediated M1-Macrophage responses. **(A)**: Representative micrographs of naïve BMDM from PDLIM2+/+ and -/- compared with BMDM cultured with LPS for 24hr (MФ-LPS). Original magnification is 10X. **(B)**: Representative micrographs showing actin staining of PDLIM2+/+ and -/- naïve BMDM and BMDM cultured with Poly I:C for 6 or 30hr. Original Magnification is 40X, scalebars represent 20µm. **(C, D)**: qPCR analyses of CXCL10 **(C)** and IL-6 **(D)** expression in PDLIM2+/+ or -/- BMDM, treated with LPS (MФ-LPS) or Poly I:C (MФ-PI:C) for 6 or 24h. Data show fold induction of gene expression compared to genotype naive (PDLIM2+/+ or -/), graphs display data from at least 4 independent experiments. **(E, F)**: ELISAs measurements of IL-1β and TNF-α secretion in cell culture supernatants from PDLIM2+/+ or -/- BMDM, naïve or stimulated with LPS (MФ-LPS) or Poly I:C (MФ-PI:C) for 24h. Data shown are all from 3 independent experiments, except MФ-PI:C; IL-1β, which shows 3 replicates from 2 independent experiments. *P<0.05. **(G)**: NO levels in supernatants from PDLIM2+/+ and -/- BMDM exposed to 10 or 100ng/ml LPS for 24h measured using the Griess assay. Data shown are from 4 independent experiments, *P<0.05. **(H)**: Transwell migration assays towards MCP-1 with naïve, or MФ-LPS- PDLIM2+/+ or -/- BMDM measured by crystal violet staining. Data represent one of three separate experiments from different macrophage cultures with similar results, normalised to the quantifications of PDLIM2+/+ migrating cells in the absence of MCP-1 set as 1 (grey bars), with fold difference of migration of cells in response to MCP-1 shown (black bars), derived from arbitrary units of crystal violet staining.

We first assessed M1 macrophage mediated by Lipopolysaccharide (LPS) for TLR4 or PI:C for TLR-3, to generate macrophage (MΦ) _LPS_ or MΦ_PIC,_ respectively. Following LPS or PI:C stimulation of primary BMDM, the morphology of naïve BMDM, with MΦ_LPS_ (**Figure 3A**) and MΦ_PIC_ were compared (**Figure 3B**). Naïve BMDM displayed a mostly elongated morphology with protrusions and membrane ruffling at ends of cells, whereas both MΦ_LPS_ (**Figure 3A**) and MΦ_PIC_ (**Figure 3B**) appeared round, with a large cytoplasm and circumferential membrane ruffling, indicative of activation (**Figure 3A**). The induction levels of M1 macrophage markers CXCL10 (**Figure 3C**), IL-6 (**Figure 3D**), CD38 (**Supplementary Figure 2A**) and IL-12b (**Supplementary Figure 2B**), were all similar in PDLIM2-/- and PDLIM2+/+ following either LPS or PI:C stimulation, indicating that the absence of PDLIM2 does not alter these M1 responses.

The functional capacity of MΦ_LPS_ and MΦ_PIC_ was assessed by measuring the production of pro-inflammatory cytokines (IL-1β, TNF-α) and iNOS. Here, we observed PDLIM2-dependent differences between LPS/TLR4 and PI:C/TLR3 responses. IL-1β secretion was lower in PDLIM2-/- MΦ_LPS_ than in PDLIM2+/+ MΦ_LPS_ (**Figure 3E**), whereas the secretion of IL-1β was similar for MΦ_PIC._ However, IL-1β RNA and protein expression were similar in MΦ_LPS_ cultures (**Supplementary Figures 2C, D**). There were no significant differences in TNF-α production (**Figure 3F**) or TNF-α gene expression (**Supplementary Figure 2E**) between any of the MΦ_LPS_ or MΦ_PIC_ cultures.

To further assess whether PDLIM2 is required for TLR3-stimulated M1 macrophage function, we measured the expression of Suppressor of Cytokine Signalling protein 3 (SOCS3) and CCL5 (41). Results showed that although there was some variation between BMDM populations isolated from different mouse cohorts, and a trend towards lower SOCS3 and CCL5 expression in PDLIM2-/- MΦ_PIC_ at 24hr, the overall differences between WT and PDLIM2-/- MΦ_PIC_, were not statistically significant (**Supplementary Figures 2F, G**).

The observed differences in IL-1β secretion in PDLIM2-/- MΦ_LPS_ (**Figure 3E**) prompted us to further investigate whether PDLIM2 is required for TLR4-mediated M1 macrophage function. Thus, we examined a classic hallmark of TLR4-macrophage signalling, iNOS protein expression and nitric oxide production. While iNOS gene and protein expression were similar in PDLIM2 +/+ and PDLIM2 -/- BMDM, (**Supplementary Figure 2H**), we observed lower Nitrite concentrations in the supernatants of PDLIM2-/- MΦ_LPS_ compared to WT controls (**Figure 3G**). This indicates lower NO generation in PDLIM2 -/- macrophages.

We next assessed the migratory capacity of PDLIM2 +/+ and -/- naïve MΦ and MΦ_LPS_ towards Monocyte Chemoattractant Protein-1 (MCP-1). We observed that naïve PDLIM2 +/+ macrophages exhibited robust migration towards MCP-1, but this was not evident in naïve PDLIM2 -/- macrophages (**Figure 3H**). However, both PDLIM2+/+ and -/- MΦ_LPS_ exhibited similar migratory capacity (**Figure 3H**), suggesting that LPS stimulation can override the lack of PDLIM2.

Finally, since it appeared that some, but not all M1-polarization responses were impaired in PDLIM2-/- BMDM, we used IFNγ as an alternative non-TLR-mediated stimulus to induce M1 polarization. The results shown in **Supplemental Figure 3** showed a similar morphology of PDLIM2-/- MΦ_IFNγ_ and PDLIM2+/+ MΦ_IFNγ_, (**Supplementary Figure 3A**), similar induction of CXCL10 and CD38 in PDLIM2+/+ and PDLIM2-/- MΦ_IFNγ_ (**Supplementary Figure 3B**), no significant differences in expression of inflammatory markers IL-1β or IL-12b (**Supplementary Figure 3C**), and similar expression levels of the IFNγR- target genes iNOS, TNF-α and IRF5 (**Supplementary Figures 3D, E**).

In summary, we conclude that although PDLIM2 is not required for TLR3 or IFNγR-mediated M1 polarization, PDLIM2 is required for the migration of naïve macrophages and for some M1 functions in response to TLR4 signalling in macrophages, including IL-1β secretion and NO production.

### PDLIM2 is required for complete adoption of the M2 macrophage phenotype

**Figure 4:**
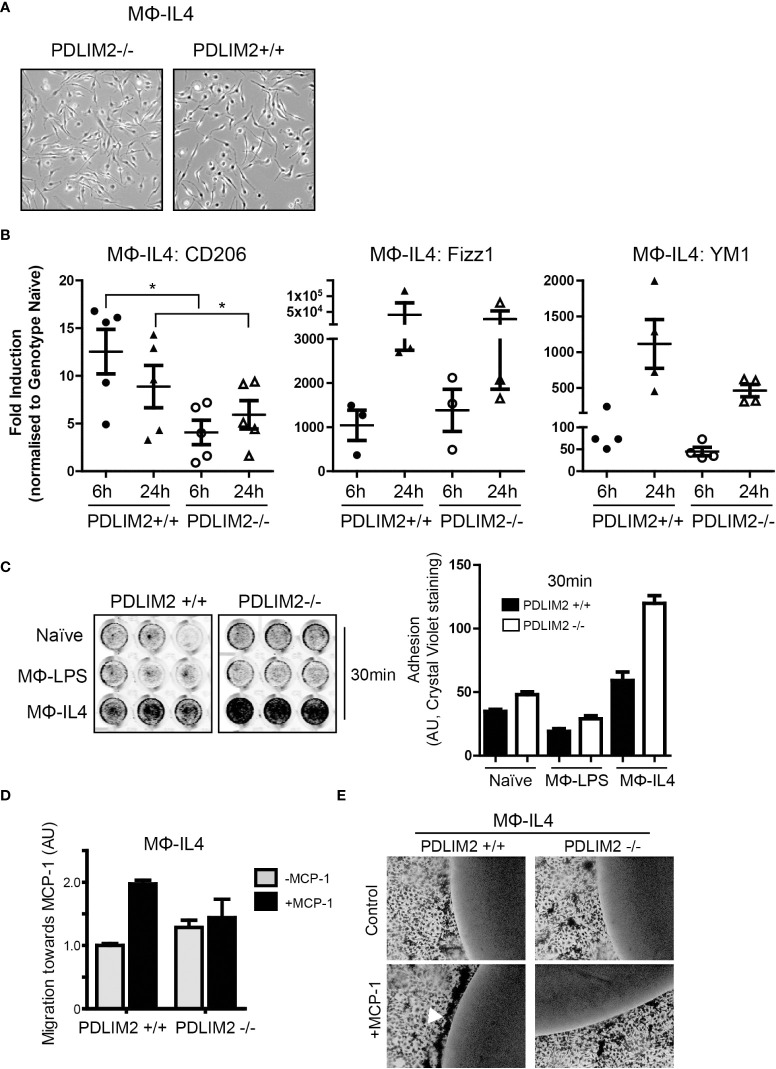
PDLIM2 is essential for complete adoption of macrophage M2-phenotype and activity. **(A)**: Representative micrographs of PDLIM2+/+ and -/- BMDM stimulated with IL-4 for 24hr (MФ-IL4). Original magnification is 10X. **(B)**: Gene expression of M2 markers CD206, Fizz1 and YM1 measured by qPCR in IL-4 stimulated PDLIM2+/+ or -/- BMDM, for 6 or 24h (MФ-IL4). Graphs show fold-induction of gene expression compared to genotype naive (PDLIM2+/+ or -/-), each data point represents an independent experiment, *P<0.05. **(C)**: Adhesion assays for PDLIM2+/+ or -/- BMDM, naïve or stimulated with IL-4 or LPS (MФ-IL4, MФ-LPS, 24h) at 30min. Representative data from one of 3 adhesion assays with similar results is shown. **(D)**: Cell migration towards MCP-1 was measured using transwell migration assays on MФ-IL-4 PDLIM2+/+ or -/- BMDM. Data is normalised to cell migration in the absence of MCP-1 (grey bars), with fold difference in response to MCP-1 shown (black bars), AU; arbitrary units of crystal violet staining. One of 3 independent experiments with similar results is shown. **(E)**: Chemotaxis agarose spot assays were performed on IL-4-stimulated BMDM from PDLIM2+/+ and -/- mice (24h). The arrow indicates BMDM migrating towards the MCP-1 gradient accumulate at the edge of the MCP-1 agarose spot. Original magnification is 10X. Also see data supplementary to D and E in **Supplementary Figure 4E**.

The next objective was to determine whether PDLIM2 is required for M2 polarization of macrophages in response to IL-4 in PDLIM2-/- and +/+ BMDM (MΦ_IL4)._ In these cultures, the morphology of PDLIM2+/+ and PDLIM2-/- MΦ_IL4_ was similar with all exhibiting elongated, polarized cell bodies with large lamellipodia at one end (**Figure 4A**). The expression of CD206 (a hallmark gene for M2 polarization) was significantly lower in PDLIM2 -/- MΦ_IL4_ than in PDLIM2+/+ MΦ_IL4_ at 24 h. YM1 and Fizz1 (markers of M2 activation), although not significantly different, consistently trended lower despite exhibiting a wide range of expression in multiple biological replicates (**Figure 4B**). However, the expression levels of two M2 activation markers: STAT-6 phosphorylation (**Supplementary Figure 4A**) and E-Cadherin (**Supplementary Figure 4B**) were similar in PDLIM2-/- and PDLIM2+/+ MΦ_IL4_.

Cell adhesion and migration are important functions in M2 macrophages and since PDLIM2 regulates cell attachment and migration in epithelial cells and differentiated THP-1 cells (7, 14, 16), we assessed both migration and adhesion in PDLIM2+/+ and PDLIM2-/- M2-MΦ_IL4_. First, cell adhesion was assessed at 30min to assess initial adhesion and at 6hr to assess ability to maintain adhesion. PDLIM2-/- naïve BMDM, MΦ_IL4_ and MΦ_LPS_ all exhibited increased adhesion to tissue culture plates within 30min compared to PDLIM2 +/+ MΦ (**Figure 4C**), which was particularly significant with PDLIM2-/- MΦ_IL4_ compared with PDLIM2+/+ BMDM. After 6hr, the differences in adhesion of naïve and LPS- PDLIM2+/+ were no longer significant, whereas the adhesion of IL4-stimulated PDLIM2-/- BMDM remained significantly higher than their PDLIM2+/+ counterparts (**Supplementary Figure 4C**).

In migration assays PDLIM2+/+ MΦ_IL-4_ migrated towards MCP-1 in transwells but, PDLIM2-/- MΦ_IL4_ exhibited little migration (**Figure 4D**). Similarly, in chemotaxis assays where cells migrate towards an MCP-1-infused agarose spot (31), PDLIM2-/- MΦ_IL-4_ cells displayed little evidence of migration (**Figure 4E**), and the migration of PDLIM2-/- naïve macrophages towards MCP-1-infused agarose was also impaired (**Supplementary Figure 4D**). These results are consistent with the enhanced adhesion capacity of these cells shown in **Figure 4C** and the impaired migration of naïve PDLIM2-/- macrophages shown in **Figure 3H**.

Overall, these results demonstrate that cell migration capacity is decreased and adhesion is increased in M2- macrophages derived from PDLIM2-/- mice, compared with PDLIM2+/+ mice. Since these are essential outputs of M2 macrophage activity, these data suggest that PDLIM2 is necessary for full M2 macrophage polarization and function.

### The capacity to activate STATs NFkB-P65 and other signalling responses in M1 or M2 polarized macrophages is not dependent on PDLIM2

M1 or M2 macrophage polarization has been associated with activation of several responses including STATs and NFκB. We therefore next asked whether there were differences in (acute/10min or long-term/up to 24hr) signalling responses induced by TLR4, TLR3, IFNγR, or IL-4R in BMDM derived from PDLIM2+/+ and -/- mice.

**Figure 5:**
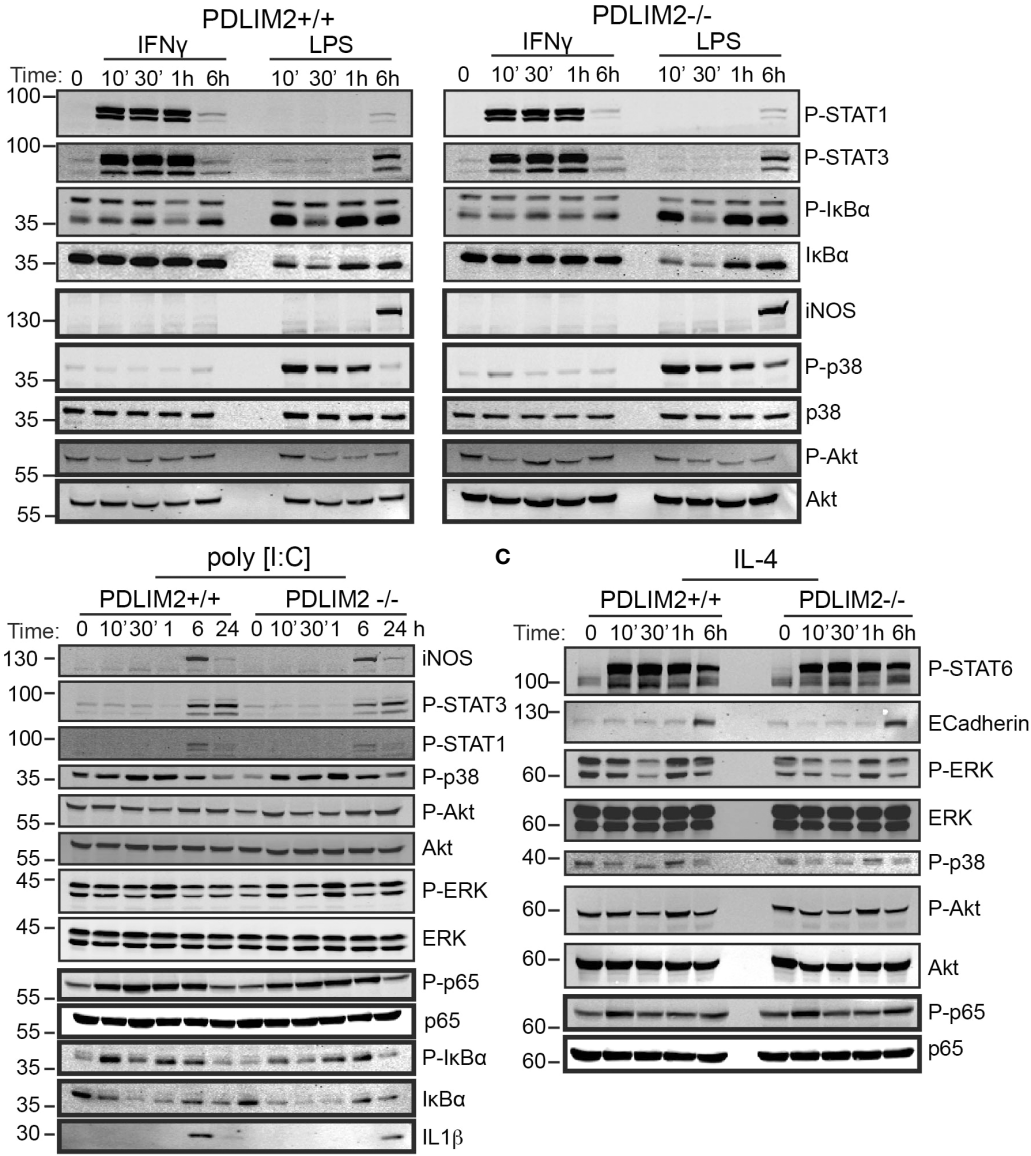
PDLIM2 is not required for activation of signalling downstream from M1 or M2 polarization receptors, including activation of STATs and NFkB-P65 in BMDM. **(A–C)**: Cell lysates from PDLIM2+/+ and -/- BMDM were cultured with IFNγ, or LPS **(A)** or PolyI:C **(B)** or IL-4 **(C)**, for the indicated times and cell lysates were prepared for western blotting with specific antibodies for the indicated proteins or phospho-proteins. The approximate protein size in KDa is shown on the left of blots.

Overall, it was observed that signalling responses were quite similar in PDLIM2-/- and +/+ BMDM for up to 6hr stimulation or at 24hr. **Figure 5A** and **Supplementary Figure 5** show the response profile for IFNγR and LPS stimulation at 10min and over time (30min up to 6hr). STAT1 and STAT3 phosphorylation were evident as early as 10min following IFNγR stimulation and sustained for up to 1hr (**Figure 5A**, **Supplementary Figure 5A, B**). However, by 6hr, levels of both phospho-STAT1 and STAT3 had returned to basal levels (**Figure 5A**), with a second peak of phosphorylation observed at 24hr (**Supplementary Figures 5C, D**). In response to LPS/TLR4 stimulation, the phosphorylation of STAT3 was substantially increased at 6hr (**Figure 5A**) and up to 24hr (**Supplementary Figure 5C**).

The phosphorylation of IκBα was induced by IFNγ in both PDLIM2+/+ and -/-BMDM at 30min and 6hr, however total IκBα levels remained unchanged up to 24hr (**Figure 5A**, **Supplementary Figure 5C**), likely because IFNγR activation would not be expected to directly engage the NFkB pathway (42), [reviewed in (43)]. Phosphorylated and total IkBα were suppressed in both PDLIM2+/+ and -/- MΦ_LPS_ after 30min, however, these increased again after 1hr (**Figure 5A**, **Supplementary Figure 5C**). The induction of iNOS in MΦ_LPS,_ and MΦ_IFNγ_ was similar for PDLIM2 +/+ and -/- BMDM at 6hr (**Figure 5A**). Levels of p38, ERK and Akt phosphorylation were also similar in PDLIM2-/- and PDLIM2+/+ MΦ_IFNγ_ (**Figure 5A**, **Supplementary Figures 5C, D**).

The profile of signalling responses to TLR3 stimulation with Poly I:C are shown in **Figure 5B**. A maximum induction of iNOS expression in both PDLIM2-/- and PDLIM2+/+ MΦ_P:IC_ was observed at 6hr (**Figure 5B**). STAT1 and STAT3 phosphorylation were increased by 6hr, although these could be detected by immunofluorescence as early as 1hr post stimulation (P-STAT1 shown in **Supplementary Figure 5E**), and phospho-STAT3 levels were sustained at 24hr (**Figure 5B**). P38 and ERK signalling were also similar in PDLIM2-/- and PDLIM2+/+ MΦ_PIC_, and phosphorylation of both proteins peaked at 1hr, whereas increased phospho-Akt was more evident following 6hr and 24hr stimulation (**Figure 5B**). Phosphorylation of NFkB p65 subunit, which started as early as 10min, and IkBα phosphorylation, which peaked at 10min PI:C exhibited similar profiles over time, and the concomitant degradation of IkBα (indicated by decreased total IkBα protein) were similar in PDLIM2-/- and PDLIM2+/+ MΦ_PIC_. The translocation of IRF3 to the nucleus was assessed as a marker of TLR3 signalling in macrophages (44). IRF3 was present in the nuclei of both PDLIM2-/- and PDLIM2+/+ MΦ_PIC_ at 6hr, and was cleared from the nucleus by 24hr (**Supplementary Figure 5F**)

The signalling response profiles of BMDM undergoing M2 polarization are shown in **Figure 5C**. These did not differ significantly between PDLIM2-/- and +/+ BMDM. STAT6 phosphorylation, E-Cadherin induction, Erk phosphorylation, p38 phosphorylation, Akt phosphorylation and p65 phosphorylation levels were all similar (**Figure 5C**, **Supplementary Figure 5C**). Phosphorylation of JNK was also evident in MΦ_IL4_ after 24hr stimulation (**Supplementary Figure 5C**). Levels of the PDLIM2 protein and its apparent phosphorylation status (mobility shift in gels) were not altered by long term (24hr) IFNγ or LPS stimulation (**Supplementary Figures 5G, H**), or by stimulation with PI:C or IL-4 over the time course of these experiments (**Supplementary Figures 5I, J**).

Overall, we conclude that PDLIM2 +/+ and -/- macrophages have a similar capacity to activate key signalling pathways in response to M1 or M2 polarizing stimuli, and this cannot account for the differences in function observed in impaired M2 functions in PDLIM2 -/- BMDM or reduced NO production in M1 macrophages.

### High PDLIM2 expression is associated with elevated M2 macrophage infiltration estimation in breast cancer datasets

The findings thus far indicated that PDLIM2 is associated with the M2 macrophage subset in breast cancers, including TNBC, and is required for the full adoption of the M2 phenotype in mouse BMDM. Since, M2 macrophage infiltration may contribute to the aggressiveness of breast cancer, we were interested to further test the relationship between PDLIM2 expression and M2 macrophage infiltration by analysing publicly available datasets for breast cancer. To do this, the METABRIC (n=1894) and TCGA-BRCA (n=1079) breast cancer datasets were interrogated using CIBERSORT and TIMER 2.0 (35, 37, 45).

**Figure 6:**
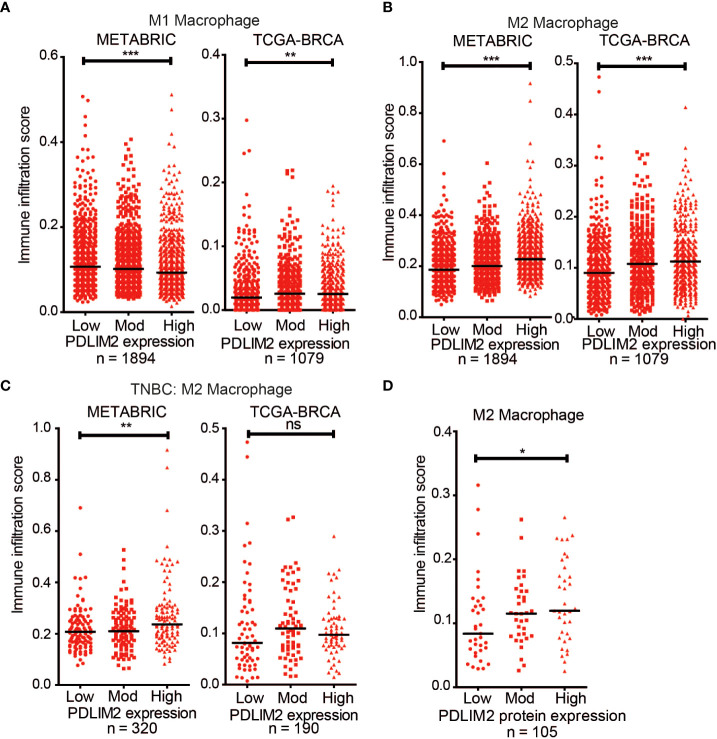
CIBERSORT deconvolution of METABRIC (n = 1894) and TCGA-BRCA (n = 1079) cohorts. Scatter plots of the immune infiltration estimation scores for **(A)** M1 macrophages and **(B)** M2 Macrophages were plotted using data generated by CIBERSORT. **(B)** Scatter plot of the M2 Macrophage infiltration estimation scores for the TNBC cases in the METABRIC (n = 320) and TCGA-BRCA (n =190) cohorts. **(D)** Scatter plot of M2 Macrophage estimation scores for TCGA-BRCA samples with proteomic data (n = 105). On all graphs the bar indicates median expression and statistical significance was assessed using a two-tailed Mann-Whitney test comparing PDLIM2_Low_ vs PDLIM2_High_ samples. *, P ≤ 0.05; **, P ≤ 0.01; ***, P ≤ 0.001.

Breast cancer cases were stratified for PDLIM2 mRNA expression into low, moderate, or high expression for analysis with the CIBERSORT-Absolute mode, which generates a score that quantitatively reflects the overall abundance of each cell type from a bulk mixture. Analysis of T cell subsets in the METABRIC and TCGA datasets did not demonstrate a consistent pattern of CD4+, CD8+, or regulatory T cell infiltration, with opposite trends observed in the two datasets (**Supplementary Figures 6B–D**). The association of naïve (M0)/M1 macrophage populations with PDLIM2 expression was also inconsistent- being negative in METABRIC and positive in TCGA datasets (**Supplementary Figure 6A**, **Figure 6A**). However, in both datasets, higher levels of M2 macrophage infiltration were clearly observed in tumours with higher PDLIM2 mRNA expression (**Figure 6B**). When TNBC cases were separately analyzed (METABRIC n=320 and TCGA n=190), there was a significantly higher estimated level of M2 macrophages observed in METABRIC tumours with higher PDLIM2 mRNA expression. In the TCGA-BRCA cohort, there was a trend towards higher M2 infiltration with higher PDLIM2 expression, but this did not reach statistical significance (**Figure 6C**).

Finally, the quantitative proteomic data that is available for a subset of the TCGA cases (n=105) through the Clinical Proteomic Tumor Analysis Consortium (CPTAC) NCI/NIH portal (47) was assessed. Analysis of the M2 macrophage estimation scores showed a significantly higher M2 infiltration in cases with higher PDLIM2 protein expression (**Figure 6D**). This result is consistent with the increased M2 macrophage infiltration observed by IHC in the breast cancer tissue (**Figure 2**).

Overall, these analyses support the role of PDLIM2 in the recruitment and differentiation of M2 macrophages in the breast cancer tumour environment and suggest that PDLIM2 expression contributes to the function of M2 TAMs in the progression of breast cancer, particularly TNBC.

## Discussion

PDLIM2 has a complex role in cancer. It can be repressed through epigenetic regulation (48), viruses, or oxidative stress (49), but is also highly expressed in more aggressive cancers (14, 50). Our previous report on high levels of PDLIM2 expression in a large proportion of TNBC cases, and its association with beta catenin activity and adhesion signalling in the tumour cells, suggested a role in TNBC aggressiveness (11). The presence of PDLIM2 in tumour stromal cells in this study raised the question whether PDLIM2 is associated with lymphocytes or macrophages, where it has known regulatory functions (8, 16) that might influence the tumour microenvironment. Here, we established that high PDLIM2 expression in breast tumour cells is associated with infiltration of an M2 macrophage population, and that PDLIM2 is required for full polarization of M2 macrophages *in vitro*. The observations from breast cancer TMAs were supported by cellular deconvolution analyses of METABRIC and TCGA-BRCA gene expression datasets, suggesting that PDLIM2 is generally associated with M2 macrophages in breast cancer.

The presence of TAMs, particularly M2 macrophages and other immune cells are now well recognized as a feature of aggressive breast cancers, and in particular TNBC (46, 51–53). A recent study in TNBC has reported that higher numbers of CD8+ T cells, γδ T cells and CD4+ memory T cells are associated with better survival in TNBC (46). On the other hand, the presence of M2-like macrophages (54), or high expression of CD163 marker in TNBC and basal-like breast cancer has been associated with a more aggressive cancer phenotype and poor survival (55, 56). M2 macrophage polarization has been linked to the production of IL-4, IL-10, TGF-beta and Granulocyte Colony Stimulating Factor (G-CSF) by tumour cells (57, 58). Moreover, co-culture of macrophages with a TNBC cell line MDA-MB-231 increases the expression of M2 markers and increases features of cancer aggressiveness (58), suggesting that TNBC cells can directly promote M2 polarization.

The presence of high PDLIM2 expression with M2 macrophage markers in the TMA suggests that PDLIM2 contributes to M2 macrophage infiltration and or polarization. We tested this further by asking whether PDLIM2 is required for either M1 or M2 polarization in mouse BMDM derived from PDLIM2 wild type and knockout mice. These studies demonstrated a requirement for PDLIM2 in M2 polarization, with the expression of key M2 markers (CD206, YM1) being reduced in the absence of PDLIM2. Interestingly, PDLIM2 was also required for the migratory capacity of naïve BMDM and for a hall mark function of inflammatory macrophages- nitric oxide production. This indicates that PDLIM2 contributes to basic macrophage functions, but is particularly important for full adoption of the M2 subtype, including cell migration and adhesion/de-adhesion. The observations with macrophage migration and adhesion are in agreement with previous observations in THP-1 derived macrophages (16), and the requirement for PDLIM2 in regulating polarized cell migration in epithelial cells and cancer cells *in vitro* and *in vivo* (14, 11).

Surprisingly, there were no distinguishable differences in activation of the STAT and NFκB signalling pathways in macrophages in response to TLR and IL-4R stimulation. This may be due to the previously described PDLIM2 actions in limiting these pathways by acting as a feedback regulator, particularly in 3D epithelial and *in vivo* models (30). This feedback regulation may not be detectable in BMDM monolayer cell cultures, which is consistent with findings in cancer cell lines, even when cell migratory capacity was inhibited by PDLIM2 suppression (11, 14, 30).

A potential role for PDLIM2 in modulating tumour immunity and chemotherapy has been proposed in lung cancer, where in mouse models PDLIM2 ectopic expression can enhance antigen presentation and T cell activity, while its suppression by reactive oxygen species promotes recruitment and pro-tumourigenic activity of alveolar macrophages (17, 49). Although this study was focused on alveolar macrophages/myeloid cells and did not address activation of M1 or M2 populations specifically, it demonstrates that PDLIM2 is critical for macrophage function and differentiation in response to a carcinogenic stimulus, and that the presence of PDLIM2 alters the profile of macrophages in the tumour microenvironment.

It will be interesting to further investigate whether PDLIM2 expression in breast cancer M2 macrophages can influence tumour immunity or therapy responses. An overall immune suppressive environment associated with fewer cytotoxic T lymphocytes in TNBC may result from interactions of M2 TAMs with tumour cells. This has been demonstrated with BRCA1-IRIS over-expressing TNBC cells that secrete GM-CSF, which facilitated the recruitment and polarization of M2 TAMs, and subsequently fewer CD8+/PD-1+ cytotoxic cells and increased CD25+/Foxp3 regulatory T cells (53, 58, 59). TNBC tumours with BRCA-1 mutations were also observed to express more PD-1 and CTLA-4 compared with BRCA-1 normal tumours. In this murine model, anti-PD-1 and CTLA-4 therapy in combination with chemotherapy reduced tumour growth (58). Although we did not observe any differences in CD8+ or Foxp3+ cell populations in breast tumours with high or low PDLIM2 expression, the association of high PDLIM2 expression with M2 macrophage infiltration, and the association of this immune subset with breast cancer aggressiveness suggests that the presence of PDLIM2 in TNBC could influence the overall tumour microenvironment.

In summary, we conclude that PDLIM2 is required for the migratory capacity of macrophages and full adoption of the M2 phenotype, and that high levels of PDLIM2 in breast cancer tumour and stroma is associated with pro-tumorigenic M2 macrophage activity, particularly in TNBC. Thus, PDLIM2 could identify tumours with M2 macrophage function and thereby increase our knowledge of immune cell interactions in the breast tumour microenvironment, which may be particularly useful for new therapy options in TNBC.

## Data availability statement

The original contributions presented in the study are included in the article/**Supplementary Material**. Further inquiries can be directed to the corresponding author.

## Author contributions

OTC, NO’S, ET, and SW conceived, performed and analyzed experiments and also prepared figures and text. NB coordinated the TMA staining, contributed to data analysis and editing of manuscript. and and RO’C conceived experiments, analyzed data and prepared the manuscript. All authors contributed to the article and approved the submitted version.

## Funding

This work was funded by Science Foundation Ireland awards 11/PI/1130 and SFI 16/IA/4505 to RO’C.

## Acknowledgments

We are grateful to our colleagues in the Cell Biology Laboratory for helpful discussions, to Dr. Silvia Melgar and the staff at the Biological Services Unit for help with BMDM studies and Ms. Elaine Gilmore for preparing IHC images. The results in **Figure 6** are in part based upon data generated by the TCGA Research Network: https://www.cancer.gov/tcga. Data used in **Figure 6D** were generated by the Clinical Proteomic Tumor Analysis Consortium (NCI/NIH).

## Conflict of interest

The authors declare that the research was conducted in the absence of any commercial or financial relationships that could be construed as a potential conflict of interest.

## Publisher’s note

All claims expressed in this article are solely those of the authors and do not necessarily represent those of their affiliated organizations, or those of the publisher, the editors and the reviewers. Any product that may be evaluated in this article, or claim that may be made by its manufacturer, is not guaranteed or endorsed by the publisher.
