# Supplementary material for: PDLIM2 is highly expressed in Breast Cancer tumour-associated macrophages and is required for M2 macrophage polarization
Source: Front Oncol. 2022 Nov 30;12:1028959. doi: 10.3389/fonc.2022.1028959 (PMC9749823; doi:10.3389/fonc.2022.1028959)
Supplement: Supplementary file 1 [file DataSheet_1.pdf]

Cox, OT et al., Supplemental Data

Supplemental Figure 1 (related to Figures 1 and 2)

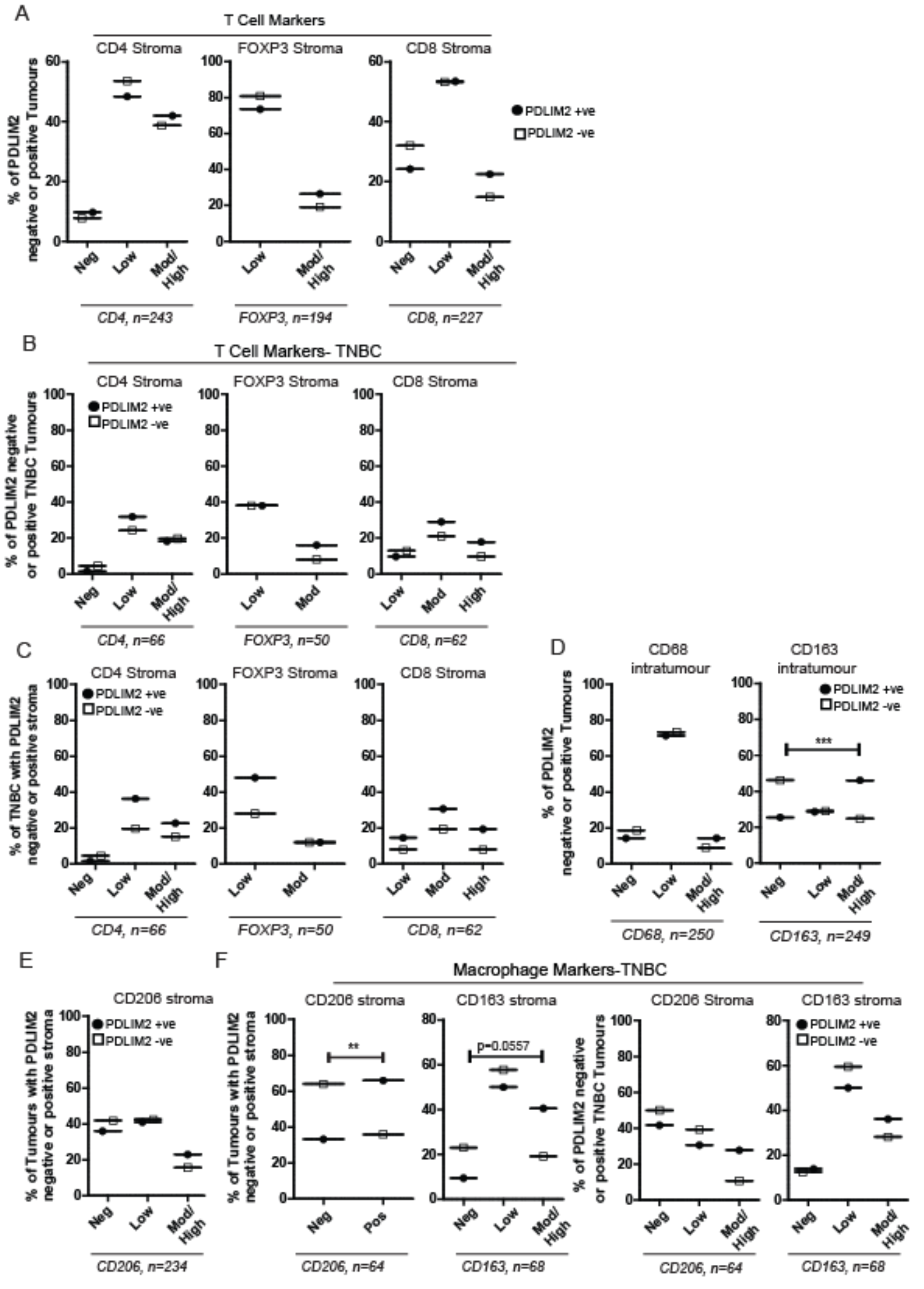

Expression of T cell and Macrophage markers in NIB TMA cohort and TNBC subset

A: Expression of T cell markers CD4, Foxp3, and CD8 scored as negative, low or moderate/high in the stroma of breast TMA cores with PDLIM2-ve or +ve tumour cells.

B, C: Expression of T cell markers in TNBC PDLIM2-ve or +ve tumours (E) and stroma (F). Data are presented as percentage of each marker expression score within all PDLIM2-ve or +ve groups (A) or TNBC subset (B,C).

D: CD68 and CD163 macrophage marker expression intra-tumourally in breast cancer tissues with PDLIM2-ve or +ve tumour cells. **E:** Expression of CD206 macrophage marker in PDLIM2-ve or +ve stroma. **F:** Expression of macrophage markers CD206 and CD163 in TNBC with PDLIM2-ve or PDLIM2+ve stroma (left panels) and tumours (right panels).

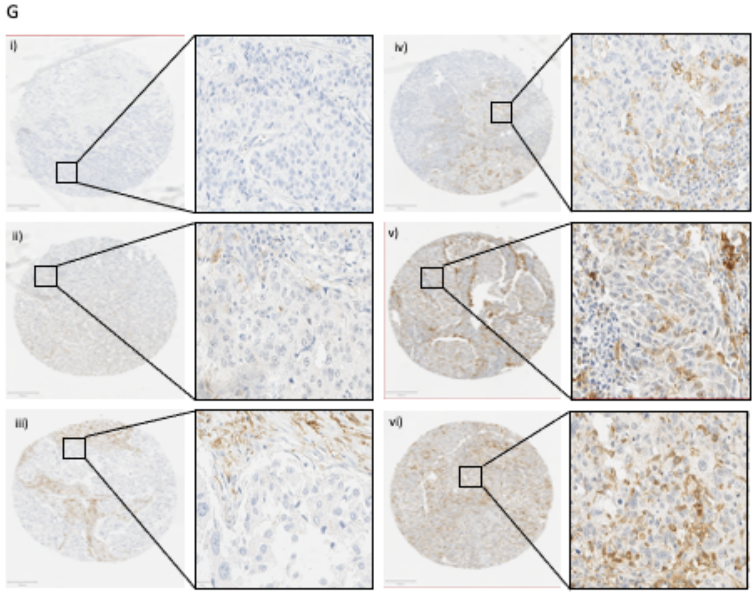

CD14 staining patterns in breast cancer.
i) negative staining
ii) negative tumour, low stroma expression
iii) negative tumour, high stroma expression
iv) low tumour, high stroma expression
v) medium tumour, high stroma expression
vi) high tumour and high stroma expression.
Magnification: Cores x 6.5, insert x 40.

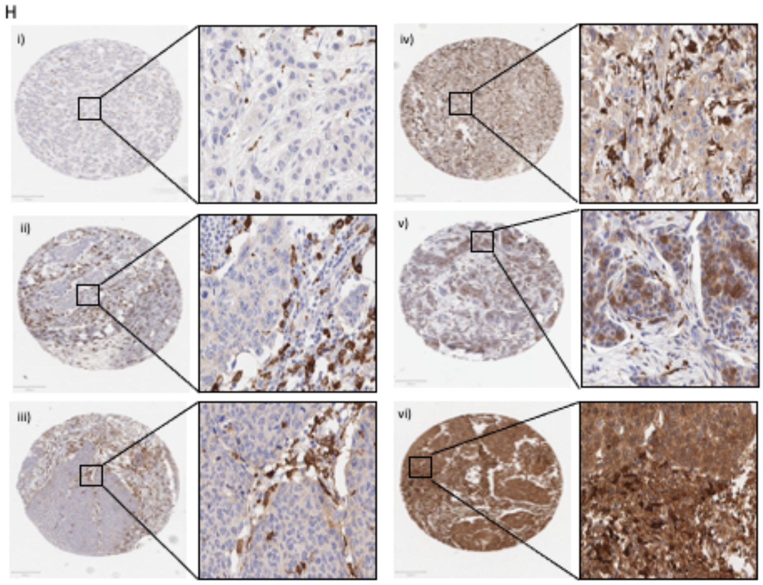

CD163 staining patterns in breast cancer.
negative tumour, low stroma
ii) negative tumour, high stroma
iii) negative tumour with infiltrating positive immune cells, high stroma
iv) low tumour, high stroma
v) medium tumour, low stroma
vi) high tumour and high stroma
Magnification: Cores x 6.5, insert x 40.

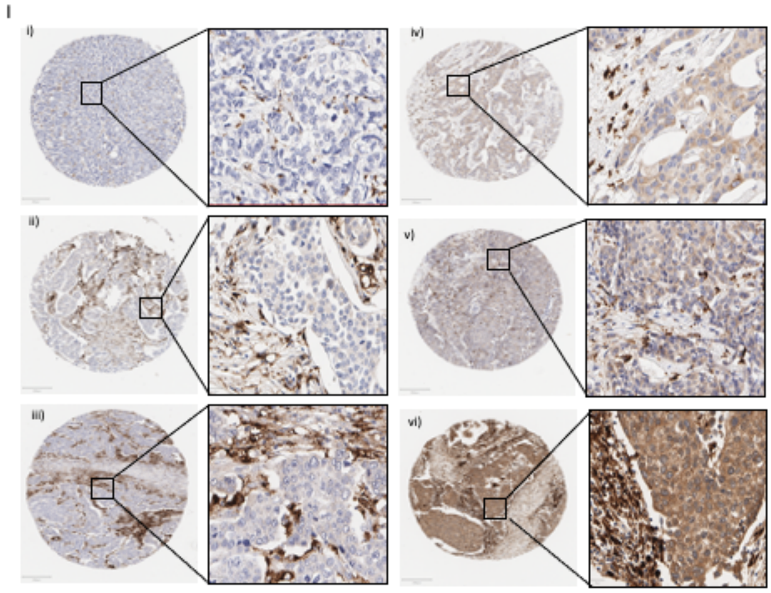

CD68 staining patterns in breast cancer. i) negative tumour, low stroma expression
ii) negative tumour, medium stroma expression
iii) negative tumour, high stroma expression
iv) low tumour, low stroma expression
v) medium tumour, medium stroma expression
vi) high tumour and high stroma expression.
Magnification: Cores x 6.5, insert x 40.

G-I Representative IHC images capturing negative and positive staining patterns for macrophage markers CD14 **(G)**, CD163 **(H)** and CD68 **(I)** in breast cancer TMA.

Supplemental Figure 2 (related to Figure 3):

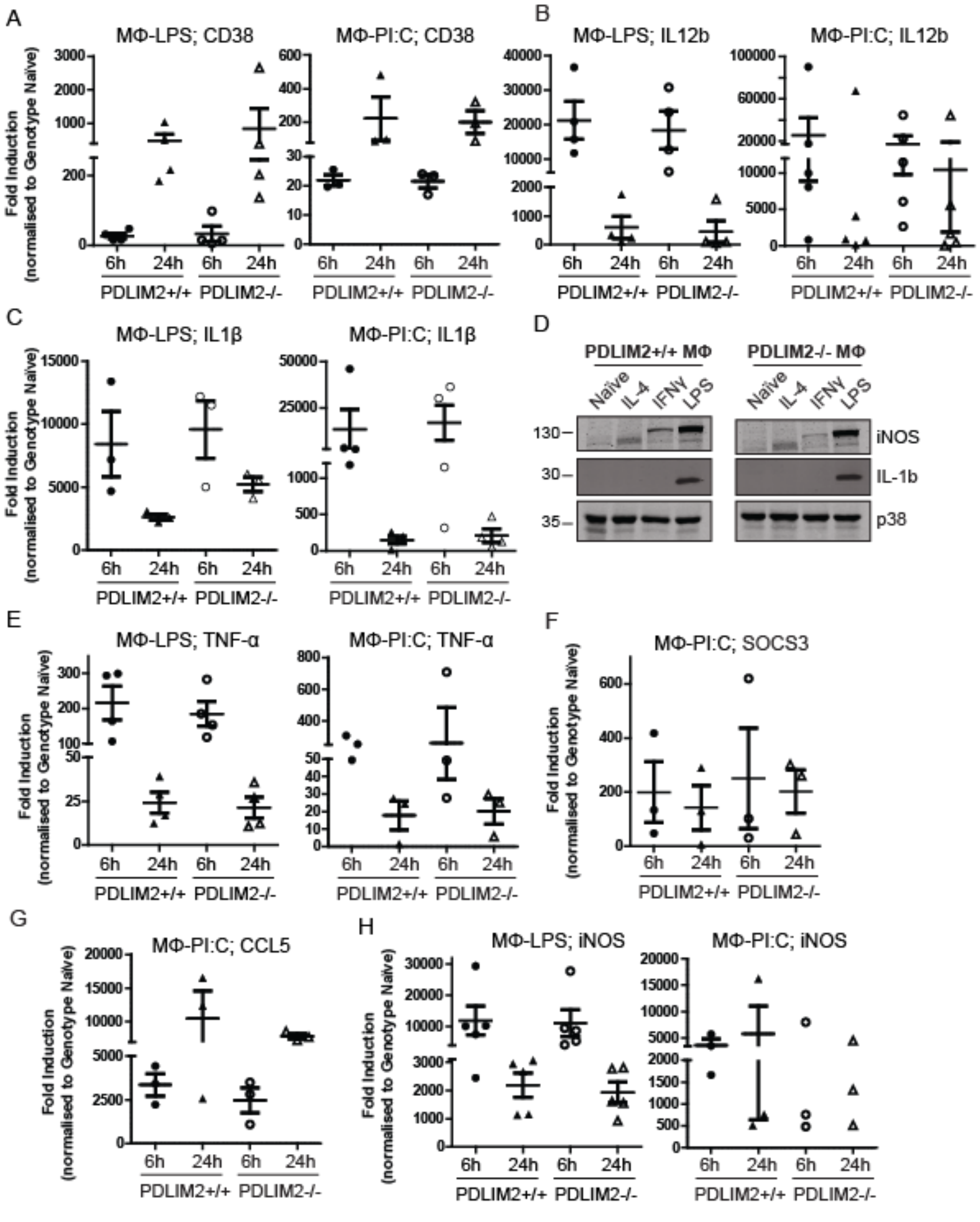

Expression of genes in PDLIM2+/+ and PDLIM2 -/- BMDM

A-C: qPCR analyses of CD38 (A) and IL-12b (B) and IL-1β gene expression in PDLIM2+/+ or -/- BMDM, stimulated with LPS (MΦ-LPS) or Poly I:C (MΦ-PI:C) for 6 or 24h. Data show fold induction of gene expression compared to genotype naive (PDLIM2+/+ or -/-), graphs display data from at least 3 independent experiments. **D:** Western blots of iNOS and IL-1β protein expression in PDLIM2+/+ or -/- BMDM treated as indicated for 24hr. p38 was used as protein loading control. Approximate protein size in KDa is indicated on the left of blots. **E:** qPCR analyses of TNFα gene expression in BMDM as for A-C. **F, G:** qPCR

analyses of SOCS3 (G) and CCL5 (H) gene expression in PDLIM2+/+ or -/- BMDM, treated with Poly I:C (MΦ-PI:C) for 6 or 24h. **H:** qPCR analyses of iNOS gene expression in BMDM. Data from 3 independent experiments with fold induction of gene expression compared to genotype naive (PDLIM2+/+ or -/-) are shown.

Supplemental Figure 3 (related to Figure 3):

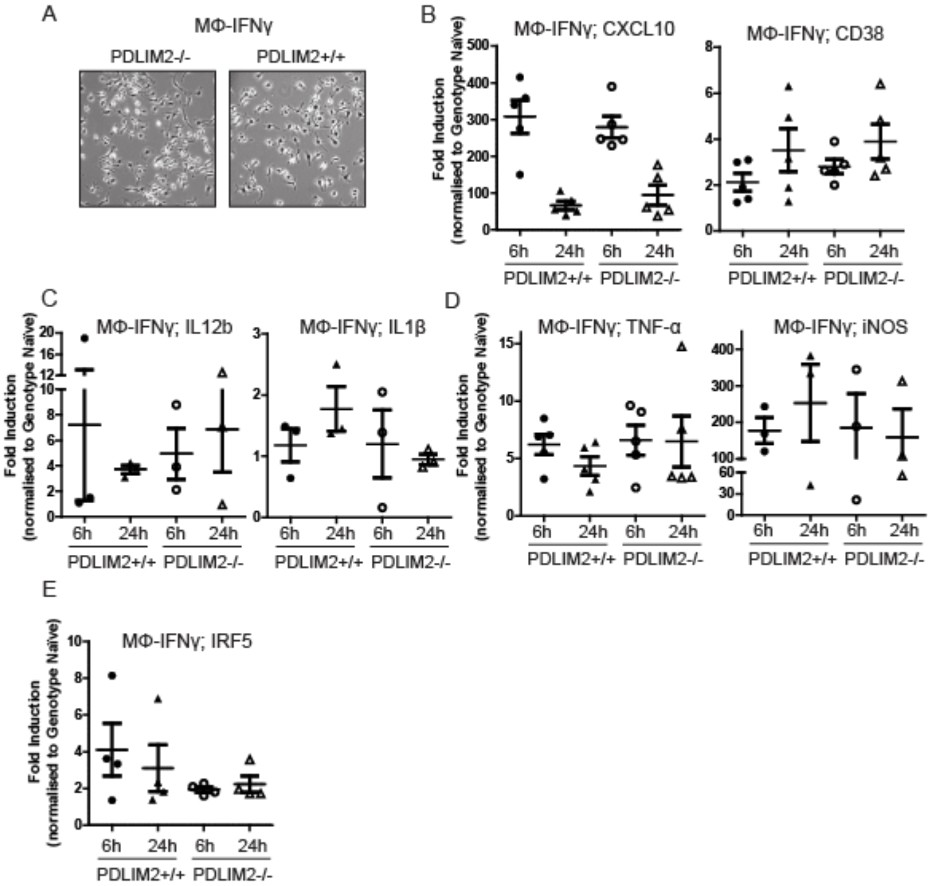

Characterization of IFNγ-stimulated BMDM

A: Representative micrographs of naïve BMDM from PDLIM2+/+ and -/- compared with BMDM stimulated with IFNγ for 24hr (MΦ- IFNγ). **B-E:** qPCR analyses of M1 polarisation markers in MΦ- IFNγ; CXCL10 and CD38 (B), IL12b and IL1b (C), TNFa and iNOS (D) and IRF5 (E) gene expression in PDLIM2+/+ or -/- BMDM, treated with IFNγ (MΦ- IFNγ) for 6 or 24h. Data show fold-induction of gene expression compared to genotype naive (PDLIM2+/+ or -/-), graphs display data from 3-5 independent experiments.

Supplemental Figure 4 (related to Figure 4):

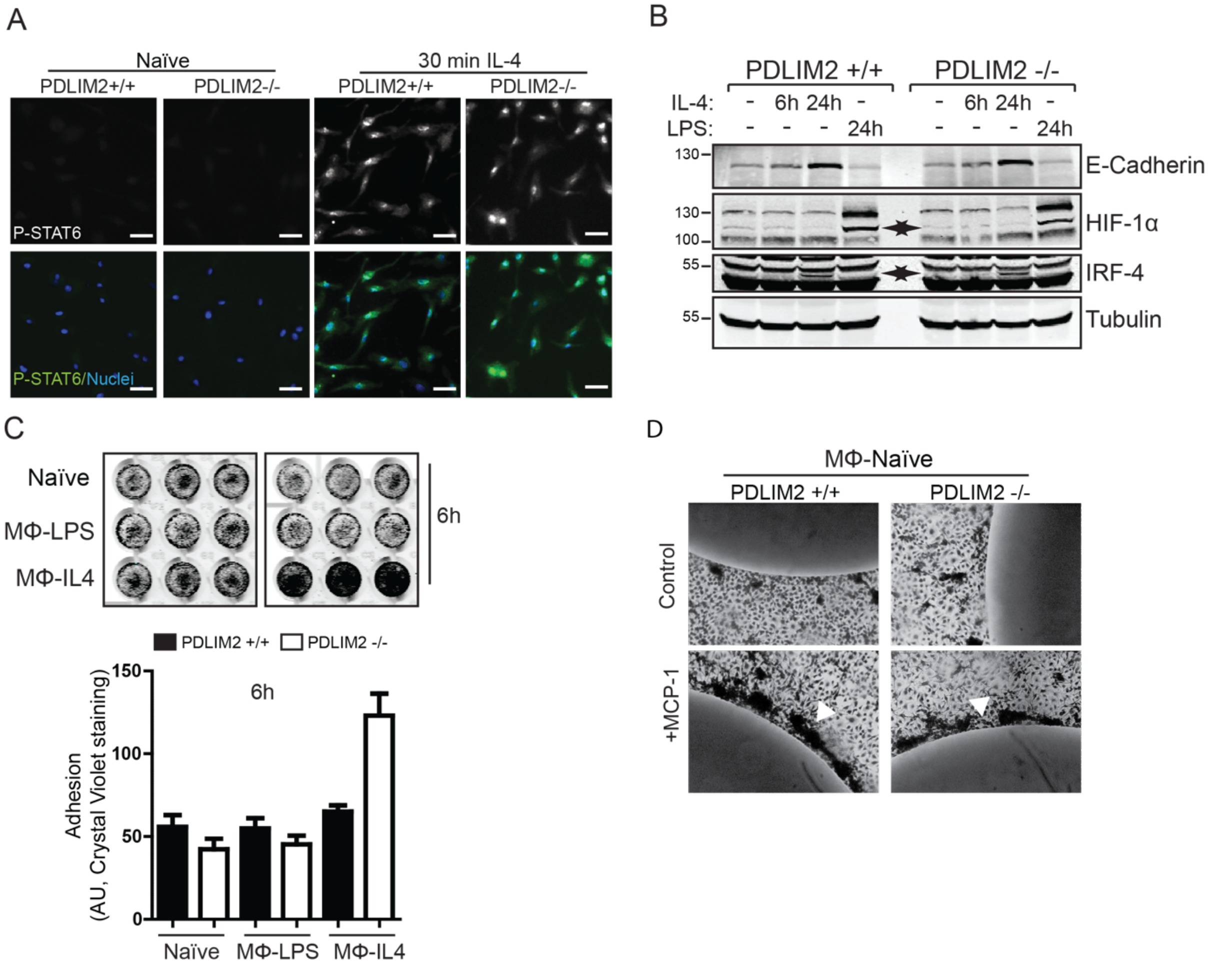

A: Immunofluorescence micrographs of BMDM from PDLIM2+/+ or -/- stained for P-STAT6 (Green, nuclei; blue) expression following 30min stimulstion with IL-4. Original Magnification is 40X, scalebars represent 20µm. **B:** Western blot of cell lysates from PDLIM2+/+ and -/- BMDM stimulated IL-4 or LPS for 6 or 24hr, probed for M2 (E Cadherin, IRF4) or M1 marker, Hif1α. Asterix indicates IRF4 and Hif1α bands. **C:** Adhesion assays with PDLIM2+/+ or -/- BMDM, naïve or stimulated with IL-4 or LPS (MΦ-IL4, MΦ-LPS, 24h) at 6hr. Graph shows quantification of adherent cells by crystal violet. **D:** Chemotaxis agarose spot motility assays with naive PDLIM2+/+ and -/- BMDM (24h). Brightfield images of cells are shown, arrows indicate BMDM that have migrated towards the MCP-1 gradient and accumulated at the edge of the MCP-1 agarose spot. Original magnification is 10X.

Supplemental Figure 5 (related to Figure 5):

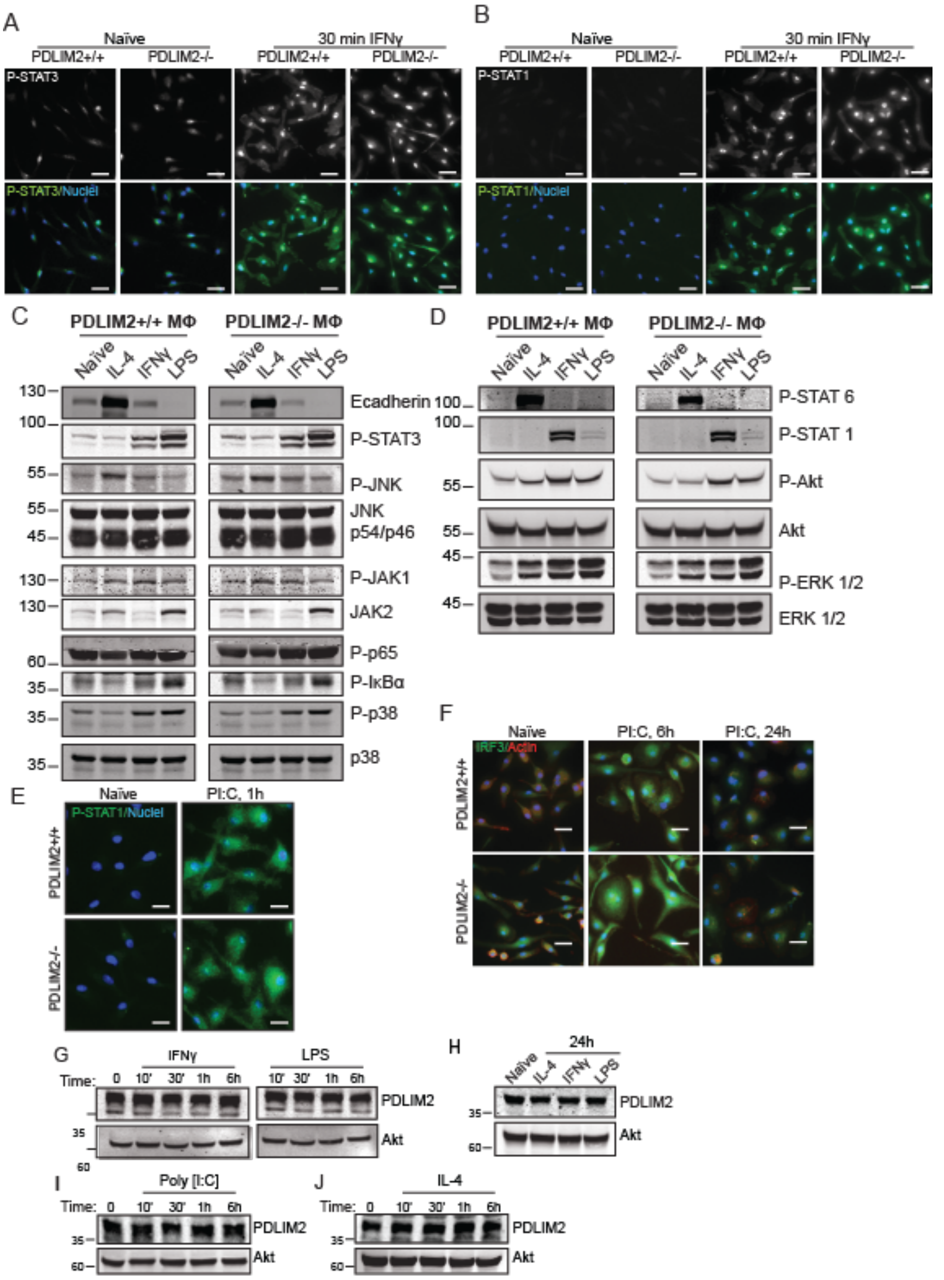

Analysis of signalling pathways in PDLIM2+/+ and PDLIM2 -/- BMDM

A, B: Immunofluorescence micrographs of naïve or IFNγ-treated (30min) PDLIM2+/+ or -/- BMDM showing nuclear expression of phospho-STAT3 (A) and Phospho-STAT1 (B) following treatment. **C, D:** Western blots of cell lysates from PDLIM2+/+ or -/- BMDM following 24hr stimulation with IL-4, IFNγ or LPS, showing expression of the indicated signalling proteins and polarisation markers. Approximate protein size in KDa is shown on the left of blots. **E, F:** Micrographs showing immunofluorescent staining of Phospho-STAT1

(E) and IRF3 (F) in PDLIM2+/+ or -/- BMDM cultured with PolyI:C for indicated times, compared with Naïve controls. Original magnification is 40X, scalebars represent 20µm.

D-J: Lysates from PDLIM2+/+ BMDM from A-C probed for PDLIM2 protein expression. Total Akt was used as loading control. Data represent at least 2 independent experiments, signalling blots are from 2 separate experiments with similar results, indicated by border of blot; thin border is one experiment, thick border is second experiment.

Supplemental Figure 6 (related to Figure 6)

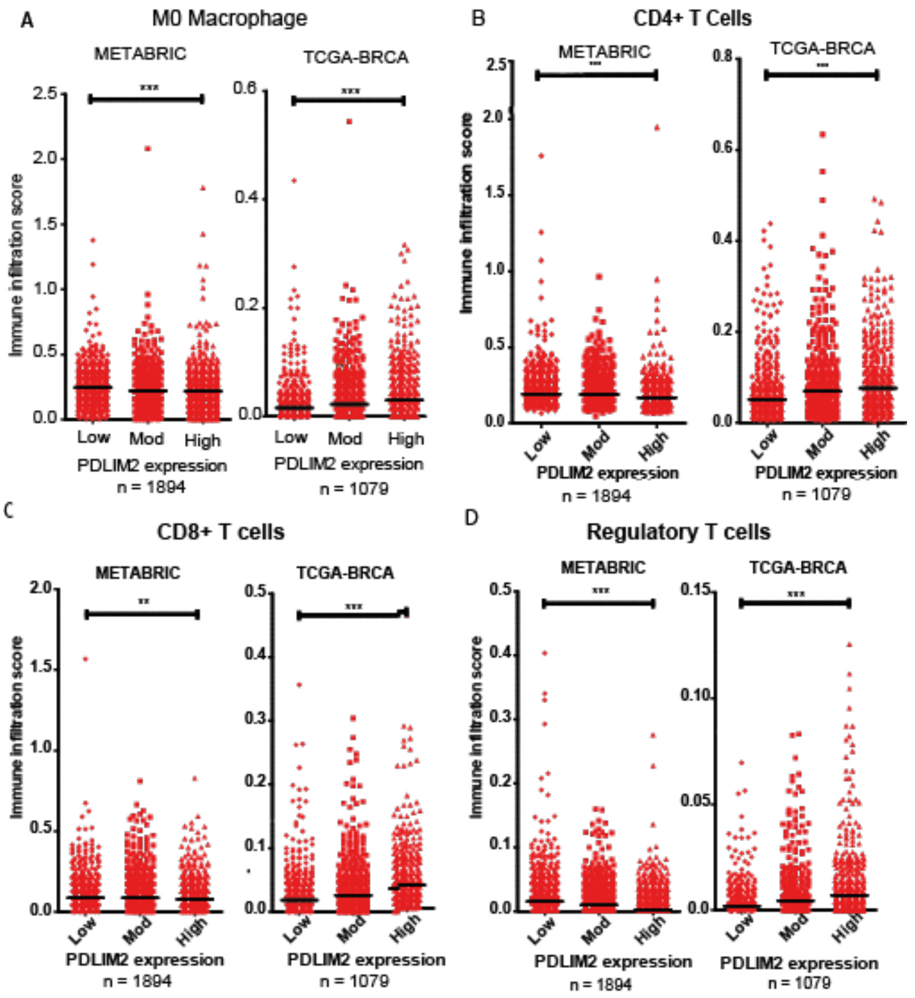

CIBERSORT deconvolution of METABRIC (n = 1894) and TCGA-BRCA (n = 1079) cohorts.

Scatter plots of the immune estimation scores for (A) M0 Macrophages, (B) all subsets of CD4+ T cells (C) CD8+ T cells, and (D) Regulatory T cells. On all graphs the bar indicates the median expression and statistical significance was assessed using a two-tailed Mann-Whitney test comparing $PDLIM2_{Low}$ vs $PDLIM2_{High}$ samples. *, $P \leq 0.05$; **, $P \leq 0.01$; ***, $P \leq 0.001$.
